# Supplementary figures and images for: Fetal death and its association with indicators of social inequality: 20-year analysis in Tacna, Peru
Source: PLoS One. 2023 Oct 5;18(10):e0292183. doi: 10.1371/journal.pone.0292183 (PMC10553794; doi:10.1371/journal.pone.0292183)

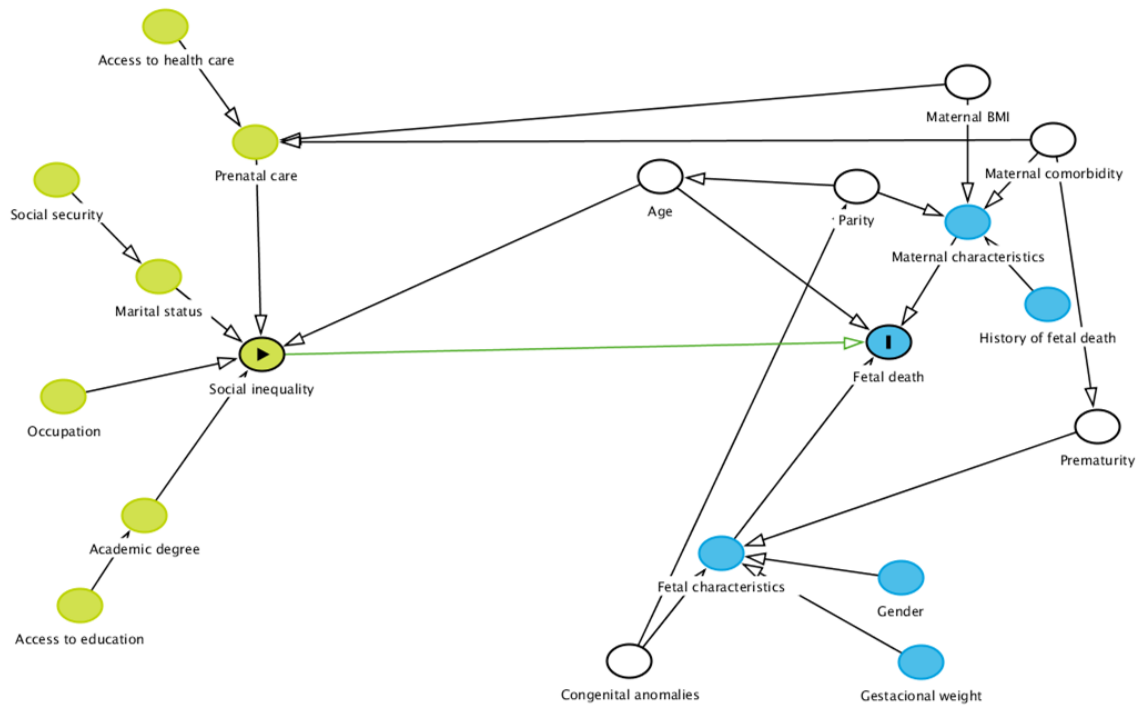

**S1 Fig:** Directed acyclic diagram to determine the possible causes of fetal death.

Supplement: S1 Fig — (PDF) [file pone.0292183.s001.pdf]
