# Supplementary material for: Fetal death and its association with indicators of social inequality: 20-year analysis in Tacna, Peru
Source: PLoS One. 2023 Oct 5;18(10):e0292183. doi: 10.1371/journal.pone.0292183 (PMC10553794; doi:10.1371/journal.pone.0292183)
Supplement: Attachment — Submitted filename: PONE-D-23-03326_reviewer.docx [file pone.0292183.s002.docx]

**Reviewer’s comments:**

**Fetal death and its association with indicators of 1 social inequality: 20-year cohort analysis in Tacna, Peru**

**Abstract**

Line 30. Sentence should state: “we conducted a retrospective cohort study”…

Line 35. Median age should accompany a range.

Line 35. Sentence should not begin with numerals. Either reword the sentence or spell out the numerical value.

Line 35-37. Please revise – grammatically incorrect.

Line 37-38. Add fetal mortality rate for 2003 and for 2016.

Line 39. All numbers less than or equal to 10 should be spelled out.

Line 44-48. Please revise the conclusion - grammatical errors.

**Introduction**

Lines 53-57: Introduction overall is meaningful but paragraphs 1 and 2 are not connected. Please revise and add in a transition.

Line 56: Sounds like you’re using the definition of stillbirth by the WHO – can you please clarify this. It’s important to clarify how fetal death is defined b/c based on the cutoffs and how it’s defined, it can be considered stillbirth or miscarriage. Table 1 in your reference (Fernanda Tavares Da Silva, 2016) lists the definition you’re using by the WHO as stillbirth. In your table 2 below you make a distinction between “no stillbirth” and “fetal death” – which can be two different things depending on the definition.

Lines 57: “The overall prevalence of fetal death is around 2%”. What year is it 2% in?

Line 60: “…Peru has not seen the same reduction as other countries…”. Are you referring to the rate of reduction here? Please clarify.

Line 61: “This is particularly concerning as reducing fetal mortality was an explicit objective of the MDG”. Should state, it is an explicit objective of the MDG.

Line 64: You mentioned “few studies” that have evaluated recent trends in fetal death. Then you mentioned two studies. “few” means three or more studies. Either add another study or you can say something like “just two studies”.

Lines 74-76: It sounds like you are looking at the association between prenatal care and education and fetal mortality. Your sentence makes it sound like you are assessing the impact of fetal mortality on prenatal care and education. Please revise the order.

**Materials and methods**

Line 89: Please refer to a “live or non-live new born” outcome as “birth” instead of “newborn”. Make sure to revise the manuscript for this discrepancy throughout.

Lines 94-99: It is unclear exactly what’s collected on the standardized data collection sheet. Is all the information collected by the obstetrician? If information is not collected at the check-up visits, how comprehensive is it at the end of pregnancy?

Line 117: “A pregnant women was considered to be at high risk…” At risk of what?

Line 127: “thinness” should be listed as “underweight”

Q1. Were women treated for comorbidities such as UTI or syphilis? What was the rate of pregnancy induced hypertension or chronic hypertension in this cohort. Adding some of these additional variables to Table 1 would be help clarify.

Q2. Did your data have any multiple gestations? If so, how was the data handled?

Q3. At what time points were the prenatal visits done? Please list gestational weeks and how many women attended vs. how much missing data there was.

**Statistical analysis**

Line 140: Please show the DAG that was used as a figure here or as a supplementary figure.

Line 141: Why are you adjusting for fetal death when assessing fetal death as an outcome? What is the significant of the “year of delivery” as a confounder? Are you implying some sort of a cohort effect – if so, please clearly discuss that in the Conclusion section.

Line 144: What graphs are you referring to? Do you mean “figures” Please revise the sentence. You do not need to say “Finally, to develop the graphs…” You can just begin the sentence by saying “we also calculated fetal mortality…” When you discuss this in the Results, you can refer to the figure numbers.

**Results**

Line 153: Report median age with range. Also please report n= with percentage of each of the variables mentioned (such as: 66.2% employed (n=?)).

Line 162: Table one should be stratified by primary exposure. When stratified by exposure, it allows assessment of potential confounding. There might be uneven distribution of these characteristics between exposed and unexposed.

Line 169: Quality of figures is very poor. Hard to read numbers. Please fix Figure 1B title (what is “prenatal controls”? did you mean to say prenatal care?). Please also refer to A B and C in the text in results when presenting numbers.

Line 187: You adjusted your model assessing the risk of fetal death with fetal death? Please clarify which variables were considered confounders. If you ran a t-test on a set of demographic variables, please show the results in Table 1.

Line 166: In your methods you mentioned prenatal checkups were at their lowest in 2011 and 2020, can you comment on why this might be under the conclusion section.

Lines 165-169: Would be meaningful to see absolute and relative change between 2011 and 2020. Please create a new table showing this change.

Lines 183-184: Table 2 shows columns “no stillbirth” and “fetal death”. Can you clarify what “no stillbirth” means?

**Conclusion**

Line 192: If fetal mortality rates vary based on context, what contexts are you referring to? Please elaborate?

Line 236: What are you referring to when you say “marked decreased in good prenatal check-ups in 2020”? In this paper, you are assessing the number of prenatal visits? What does “good” refer to her?

Lines 247-249: These lines should be merged with the paragraph before? It does not need its own paragraph.

Lines 250-257: Limitations section is very weak. There is also collection of data retrospectively – mentioned in the methods section. Can you please comment on the impact of data collected at the end of pregnancy vs. at each antenatal visit.

There is nothing on the strengths of the study, please add.

Lines 259-261: It is very unclear whether fetal mortality trends you observed in your study are in line with the national trends or not. In lines 203-206 in the Results section, you mention: These measures have impacted fetal mortality at the national level, with an estimated 2% decrease each year between 2000 and 2015 [24]. However, in our study, we did not observe a clear trend in the reduction of fetal mortality.” But in the conclusion here, you mention: “In conclusion, in the hospital evaluated, fetal mortality was similar to the Peruvian rate…”.

Please also revise this sentence per grammatical errors.

Overall: Writing needs to be revised. Sentences and ideas are not clear. Analysis needs revision especially assessing confounders.
