# Supplementary material for: Fetal death and its association with indicators of social inequality: 20-year analysis in Tacna, Peru
Source: PLoS One. 2023 Oct 5;18(10):e0292183. doi: 10.1371/journal.pone.0292183 (PMC10553794; doi:10.1371/journal.pone.0292183)
Supplement: Attachment — Submitted filename: Reviewers comments.docx [file pone.0292183.s004.docx]

I reviewed the study titled “Fetal death and its association with indicators of social inequality: 20-year analysis in Tacna, Peru”.

The manuscript has addressed the previous comments satisfactorily. There are few comments that should be addressed before the paper goes for publication:

1. Lines 266-267. Authors mentioned that reduced rate of fetal death in recent years “which may be due to advancements in healthcare practices, medical technology, and prenatal care over time.”

It will be better if authors can comment on the change in literacy level or ANC care seeking in the same years and see if it is aligned with the reduction in fetal mortality. It is suggested because if a significant change can be brought by improving the system delivery alone then how much incremental change can be caused by addressing the social determinants.

2. Line 290- In limitations, “Additionally, the study was only conducted in one institution, which makes the results unrepresentative of the entire Peruvian population.”

It can be rephrased as “Additionally, the study was only conducted in one institution, hence the results are not generalizable to entire Peruvian population.”

3. Lines 292-293 “It also has a large sample size and a large amount of information about the variables of interest, which helps to have a better vision of reality.”

It needs rephrasing. It sounds like a non-scientific statement. Please rephrase and talk about study power and accurate results instead of reality as reality is a subjective term.

4. Line 298. “A more recent year of delivery was associated with lower fetal mortality.”

Add some description why it happened. Just the statement in the conclusion is not informative for the readers.
