# Supplementary material for: Fetal death and its association with indicators of social inequality: 20-year analysis in Tacna, Peru
Source: PLoS One. 2023 Oct 5;18(10):e0292183. doi: 10.1371/journal.pone.0292183 (PMC10553794; doi:10.1371/journal.pone.0292183)
Supplement: Attachment — Submitted filename: Answer plos one - Final.docx [file pone.0292183.s005.docx]

**Response to reviewers:**

We appreciate your review. Your comments enhance the depth of our manuscript. Comments are answered below:

1. Lines 266-267. Authors mentioned that reduced rate of fetal death in recent years “which may be due to advancements in healthcare practices, medical technology, and prenatal care over time.”

It will be better if authors can comment on the **change in literacy level or ANC** care seeking in the same years and see if it is aligned with the reduction in fetal mortality. It is suggested because if a significant change can be brought by improving the system delivery alone then how much incremental change can be caused by addressing the social determinants.

- Thank you, we follow your suggestion and modify the wording, as follows: “In the adjusted analysis, we found that a more recent year of delivery was associated with lower fetal mortality. This suggests a cohort effect, which may be due to the reduction in the percentage of illiteracy, which decreased from 8.3% to 0.3% in Tacna, Peru, between 2000 and 2015 [35,36]. On the other hand, the percentage of pregnant women with three controls prenatal or less decreased from 7% to 4.2% between the years 2000 and 2015, showing improved access to health care [35,36].”

2. Line 290- In limitations, “Additionally, the study was only conducted in one institution, which makes the results unrepresentative of the entire Peruvian population.” It can be rephrased as “Additionally, the study was only conducted in one institution, hence the results are not generalizable to entire Peruvian population.”

- Thank you, we made the suggested modification as follows: “Additionally, the study was only conducted in one institution, hence the results are not generalizable to entire Peruvian population.”

3. Lines 292-293 “It also has a large sample size and a large amount of information about the variables of interest, which helps to have a better vision of reality.”

It needs rephrasing. It sounds like a non-scientific statement. Please rephrase and talk about study power and accurate results instead of reality as reality is a subjective term.

- Thank you, we follow your suggestion and modify the wording, as follows: “Furthermore, the large sample size and the large amount of information on the variables of interest provide greater statistical power to our study.”

4. Line 298. “A more recent year of delivery was associated with lower fetal mortality.”

Add some description why it happened. Just the statement in the conclusion is not informative for the readers.

- Thank you, we follow your suggestion and modify the wording, as follows: “A more recent year of delivery was associated with lower fetal mortality, probably due to reduced illiteracy and increased access to health care between 2000 and 2015.”
