# Supplementary material for: Fetal death and its association with indicators of social inequality: 20-year analysis in Tacna, Peru
Source: PLoS One. 2023 Oct 5;18(10):e0292183. doi: 10.1371/journal.pone.0292183 (PMC10553794; doi:10.1371/journal.pone.0292183)
Supplement: Attachment — Submitted filename: Answer plos one 3.docx [file pone.0292183.s006.docx]

**Response to reviewers:**

We appreciate your review. Your comments enhance the depth of our manuscript. Comments are answered below:

Reviewer #2: Thanks for submitting the revised manuscript. All the comments have been addressed satisfactorily except one. Authors revised the statement to “Furthermore, the large sample size and the large amount of information on the variables of interest provide greater statistical power to our study.”

Unless you do a post-hoc power calculation, it cannot be claimed. It is suggested to calculate the power, if it has not been done at the time of sample size estimation and report it. For e.g. the large sample size provided the sufficient statistical power (80%) to our study. Please note that power is only related to sample size and not number of variables or large amount of information.

Thank you, we follow your suggestion. and calculated the statistical power of our study. We detail this information in the methodology section as follows: “We used the study by Kayode G. et al. [15] to calculate statistical power. In this research, the exposure variable was pregnants not educated. Of the pregnant women with adequate education group, 3.4% had fetal death during follow-up, while those without education had 6.4% fetal death. Likewise, a non-exposed/exposed ratio of 1.75 (3284/1866) was reported. With these parameters, a confidence level of 95% and 67,908 participants in the sample of this study, a statistical power of 99.9% was calculated.” We also detail this information in the Discussion section, on the strengths of the study, and modify the wording, as follows: “…Furthermore, the large sample size provided the sufficient statistical power (99.9%) to our study.”

Additionally, the information added on this and the previous review was properly cited and added to the bibliography (No. 15, 36 and 37). Consequently, we update the reference list.

We consider that after following your recommendations, our study has improved its quality.

Thank you.
